# Integrating Cardiopulmonary Exercise Testing and Stress Echocardiography to Predict Clinical Outcomes in Hypertrophic Cardiomyopathy

**DOI:** 10.3390/jcm14207231

**Published:** 2025-10-14

**Authors:** Geza Halasz, Paolo Ciacci, Raffaella Mistrulli, Guido Giacalone, Aurora Ferro, Giulio Francesco Romiti, Fiammetta Albi, Domenico Gabrielli, Federica Re

**Affiliations:** 1 Division of Cardiology, Cardio-Thoracic and Vascular Department, Azienda Ospedaliera San Camillo Forlanini, 00152 Rome, Italy; 2 Fondazione Policlinico Universitario Campus Bio-Medico, Via Alvaro del Portillo, 200, 00128 Rome, Italy; 3 Department of Clinical and Molecular Medicine, Sapienza University of Rome, 00185 Rome, Italyguido.giacalone.95@gmail.com (G.G.); 4 Department of Translational and Precision Medicine, Sapienza University of Rome, 00185 Rome, Italy

**Keywords:** hypertrophic cardiomyopathy, cardiopulmonary exercise testing, stress echocardiography, risk stratification

## Abstract

**Background:** Hypertrophic cardiomyopathy (HCM) is a heterogeneous myocardial disease in which conventional prognostic models, primarily focused on sudden cardiac death, often fail to identify patients at risk of clinically relevant events such as heart failure progression or rehospitalization. Cardiopulmonary exercise testing (CPET) quantifies functional capacity, while stress echocardiography (SE) provides mechanistic insights into exercise-induced hemodynamic changes. Their combined application (CPET–SE) may enhance risk stratification in patients with HCM. **Methods:** In this retrospective study, 388 patients with obstructive and non-obstructive HCM (mean age 48 ± 15 years, 63.1% male) underwent baseline CPET–SE between 2010 and 2022 and were followed for a median of 7.4 years [IQR 4.3–10.2]. Echocardiographic parameters were assessed at rest and peak exercise, and CPET indices included peak oxygen consumption (pVO_2_), ventilatory efficiency, and anaerobic threshold. The primary outcome was a composite of heart failure hospitalization or progression to end-stage HCM. **Results:** Over a median follow-up of 7.4 years, 63 patients (16.2%) experienced an event of the primary outcome. Patients who developed a primary outcome had greater left atrial diameter (45.0 vs. 41.0 mm, *p* < 0.001) and indexed volume at rest (36.4 vs. 29.0 mL/m^2^, *p* < 0.001), with further dilation during stress (*p* = 0.046); increased LV wall thickness (*p* = 0.001); higher average E/e′ at rest and during stress (*p* ≤ 0.004); and higher pulmonary artery systolic pressure at rest (*p* = 0.027) and during stress (*p* = 0.044). CPET findings included lower pVO_2_ (16.0 vs. 19.5 mL/kg/min, *p* = 0.001), reduced % predicted pVO_2_ (*p* = 0.006), earlier anaerobic threshold (*p* = 0.032), impaired ventilatory efficiency (*p* = 0.048), and chronotropic incompetence (*p* < 0.001) in patients who experienced a primary outcome. Multivariable analysis identified dyslipidemia (OR 2.58), higher E/e′ (OR 1.06), and lower pVO_2_ (OR 0.92) as independently associated with the primary outcome. **Conclusions:** CPET–SE provided a comprehensive evaluation of patients with HCM, associating aerobic capacity to its hemodynamic determinants. Reduced pVO_2_ showed the strongest association with adverse outcomes, while exercise-induced diastolic dysfunction and elevated pulmonary pressures identified a high-risk phenotype. Incorporating CPET–SE into longitudinal management of patients with HCM may enable earlier detection of physiological decompensation and guide personalized therapeutic strategies.

## 1. Introduction

Hypertrophic cardiomyopathy (HCM) is a primary myocardial disease characterized by unexplained left ventricular hypertrophy in the absence of abnormal loading conditions such as hypertension or valvular disease [1]. About 30–40% of cases have a clear genetic basis, most commonly involving autosomal dominant mutations in sarcomeric protein genes [2]. The clinical expression of HCM is highly heterogeneous, reflecting the interplay of genetic, structural, and functional factors.

Traditionally, the risk of sudden cardiac death (SCD) has been the primary concern in prognostic evaluation, supported by established risk algorithms and criteria for implantable cardioverter-defibrillator (ICD) implantation. However, mortality-based endpoints do not fully capture the disease burden as a significant proportion of patients develop progressive heart failure despite preserved ejection fraction, often culminating in advanced therapies such as heart transplantation. In addition, atrial fibrillation and other arrhythmias are frequent and may anticipate or accelerate clinical decline [3,4]. These limitations highlight the need for tools capable of predicting not only SCD but also non-fatal yet clinically meaningful outcomes, including worsening heart failure, rehospitalization, and transition to end-stage disease.

Cardiopulmonary exercise testing (CPET) provides an integrated assessment of cardiovascular, pulmonary, and muscular responses during effort, offering an objective measure of functional capacity [5,6]. Several CPET-derived parameters have been validated as markers of disease severity and progression in HCM [7]. In particular, reduced peak VO_2_—defined as <80% of predicted for age and sex or <14 mL/kg/min—has consistently emerged as a robust predictor of adverse outcomes, including clinical deterioration, cardiovascular death, and need for transplantation [7,8,9,10,11,12]. Despite this evidence, CPET is still underutilized in the longitudinal management of HCM.

Stress echocardiography (SE) complements CPET by identifying exercise-induced mechanisms of limitation, such as left ventricular outflow tract obstruction (LVOTO), systolic anterior motion (SAM) of the mitral valve, impaired diastolic reserve (E/e′), exercise-induced pulmonary hypertension, ischemia, and worsening mitral regurgitation [13,14,15,16,17]. For example, an exercise-induced LVOT gradient ≥50 mmHg is independently associated with symptom progression and heart failure hospitalization.

When performed in combination, CPET–SE integrates the prognostically validated metrics of CPET with the mechanistic insights of SE, enabling simultaneous evaluation of functional capacity and its underlying determinants. This comprehensive approach has the potential to refine risk stratification, predict progression to heart failure, and guide long-term management in HCM. Based on these premises, we sought to evaluate the prognostic utility of combined CPET–SE in a single-center cohort of patients with HCM, focusing on clinically meaningful endpoints such as rehospitalization and progression to end-stage disease.

## 2. Materials and Methods

### 2.1. Population and Clinical Assessment

We retrospectively evaluated patients with hypertrophic cardiomyopathy (HCM) who were referred to and followed at our dedicated HCM outpatient clinic between 2010 and 2023. Inclusion criteria were age ≥ 18 years, a diagnosis of HCM established according to the most recent guidelines [14], left ventricular ejection fraction (LVEF) > 50%, and the ability to perform cardiopulmonary exercise testing (CPET). Patients with a history of coronary artery disease (CAD) or with alternative diagnoses such as cardiac amyloidosis or Fabry disease (i.e., phenocopies) were excluded. The study complied with the Declaration of Helsinki, and written informed consent was obtained from all participants.

The primary endpoint was the first occurrence of a composite outcome including:

(a) Heart failure (HF) hospitalization, defined as an unplanned, overnight hospital admission with a primary diagnosis of HF requiring intravenous loop diuretics, vasodilators, or inotropes;
(b) Progression to end-stage HCM, operationally defined as transition to the advanced phase characterized by new-onset left ventricular systolic dysfunction with LVEF < 50% persisting for ≥3 months in the absence of alternative causes (e.g., acute myocardial infarction, significant valvular disease), and/or fulfillment of advanced-therapy criteria including listing for heart transplantation or implantation of durable left ventricular assist device (LVAD) due to progressive pump failure. This definition is consistent with contemporary descriptions of the end-stage phenotype and prior cohort studies in HCM [14].

All patients underwent a comprehensive clinical and cardiological evaluation, including detailed medical history, assessment of symptoms and functional status according to the New York Heart Association (NYHA) classification, a standard 12-lead electrocardiogram (ECG), 24 h ambulatory ECG (Holter) monitoring, transthoracic two-dimensional and Doppler echocardiography, cardiac magnetic resonance imaging (CMR), and combined transthoracic echocardiography with cardiopulmonary exercise testing (CPET-SE). Results of genetic testing for HCM-related pathogenic variants, when available, were also collected.

### 2.2. Cardiopulmonary Exercise Test and Stress Echocardiography

After baseline echocardiography, patients underwent a symptom-limited CPET-SE performed on an upright cycle ergometer with a ramp protocol of incremental load at 10 W/min. A respiratory exchange ratio (RER) > 1.05 was required to define maximal effort; patients who did not achieve this threshold were excluded from the analysis. Exhaled gases were continuously sampled using a mouthpiece-mounted sensor and analyzed to measure oxygen consumption (VO_2_), carbon dioxide production (VCO_2_), and minute ventilation (VE). The following exercise parameters were recorded, peak oxygen consumption (pVO_2_), ventilatory efficiency (VE/VCO_2_ slope), and oxygen pulse (VO_2_/heart rate), according to standard methodology [18,19].

Echocardiographic parameters were acquired at three predefined time points: at rest, at peak exercise, and after one minute of recovery. Two-dimensional imaging included standard acquisitions at rest and apical four-, two-, and three-chamber views during exercise to evaluate global and regional left ventricular systolic function. Doppler measurements included transvalvular aortic velocity, left ventricular outflow tract (LVOT) velocity–time integral for stroke volume calculation, peak LVOT velocity for estimation of LVOT gradient, mitral inflow and tissue Doppler imaging of the mitral annulus (e′ velocity) to assess diastolic function, and tricuspid regurgitation velocity. M-mode was used to measure tricuspid annular plane systolic excursion (TAPSE) as an index of right ventricular systolic function [20,21]. All imaging and CPET data were digitally recorded and subsequently analyzed offline by experienced operators.

### 2.3. Statistical Analysis

The distribution of continuous variables was assessed using the Shapiro–Wilk test. Continuous data are presented as mean ± standard deviation (SD) or as median with interquartile range [IQR], depending on distribution. Categorical variables are expressed as absolute frequencies and percentages. Comparisons between groups were performed using the unpaired Student’s *t*-test or Mann–Whitney U test for continuous variables, and the Chi-squared or Fisher’s exact test for categorical variables. Univariate logistic regression was applied to identify variables associated with the primary outcome. Variables with a statistically significant association (*p* < 0.05) were subsequently entered into a multivariable logistic regression model to determine independent predictors. Results are reported as odds ratios (OR) with 95% confidence intervals (CI). A two-sided *p*-value < 0.05 was considered statistically significant. All statistical analyses were performed using R software (version 4.3.1).

## 3. Results

### 3.1. Clinical Characteristics

Baseline characteristics are summarized in Table 1. A total of 388 patients were included (mean age of 48 ± 15 years 63.1% male). Most individuals were mildly symptomatic at baseline, with 92.9% in NYHA class I or II. Cardiovascular comorbidities included hypertension in 39.4% of patients, dyslipidemia in 30.2%, and atrial fibrillation in 13.1%. Patients who experienced adverse events during follow-up were significantly older (51 ± 13 vs. 47 ± 15 years, *p* = 0.047) and had a higher prevalence of hypertension (54.0% vs. 36.6%, *p* = 0.015), dyslipidemia (50.8% vs. 26.2%, *p* < 0.001), and atrial fibrillation (31.7% vs. 9.5%, *p* < 0.001) compared to those without events. They also reported dyspnea more frequently (66.7% vs. 46.2%, *p* = 0.004), and were less often in NYHA class I (20.6% vs. 51.2%, *p* < 0.001), while more commonly in NYHA class II (65.1% vs. 43.1%, *p* = 0.002). The ESC risk score for sudden cardiac death was also significantly higher in the adverse event group (4.9 ± 2.6 vs. 3.5 ± 2.2, *p* < 0.001), reflecting a greater baseline risk burden. Regarding pharmacological treatment, patients in the adverse event group were more frequently treated with beta-blockers (73.0% vs. 58.5%, *p* = 0.04), aspirin (30.2% vs. 12.9%, *p* = 0.001), ACE inhibitors (38.1% vs. 13.5%, *p* < 0.001), anticoagulants (30.2% vs. 9.3%, *p* < 0.001), amiodarone (20.6% vs. 6.2%, *p* < 0.001), and mineralocorticoid receptor antagonists (12.7% vs. 1.8%, *p* < 0.001). Diuretics were also more commonly used in the event group (43.5% vs. 14.9%, *p* < 0.001).

### 3.2. Echocardiographic Findings

Structural and functional markers of disease severity were more pronounced in the adverse event group (Table 2). They showed greater maximal LV wall thickness (22.0 [19.0–25.0] mm vs. 20.0 [17.0–23.0] mm, *p* = 0.001), larger left atrial diameter (45.0 [41.0–52.0] mm vs. 41.0 [37.0–45.0] mm, *p* < 0.001), and higher resting LA volume index (36.4 [29.8–49.0] vs. 29.0 [23.0–37.0] mL/m^2^, *p* < 0.001). LA dilation was further accentuated during stress (43.0 [0.0–66.0] vs. 26.0 [0.0–44.0] mL/m^2^, *p* = 0.046). Diastolic function was more impaired in patients with events, with higher average E/e′ ratios both at rest (11.4 [9.0–17.3] vs. 9.8 [7.7–12.6], *p* = 0.004) and during stress (11.2 [9.6–15.5] vs. 9.9 [8.1–12.3], *p* = 0.001). Pulmonary artery systolic pressure was also higher at rest (25.0 [15.0–32.0] vs. 20.0 [11.5–26.0] mmHg, *p* = 0.027) and during stress (37.0 [25.7–54.0] vs. 34.0 [25.0–45.0] mmHg, *p* = 0.044). No differences were observed in LV volumes, LVEF, or resting/provoked LVOT gradients.

### 3.3. CPET Results

**Figure 1:**
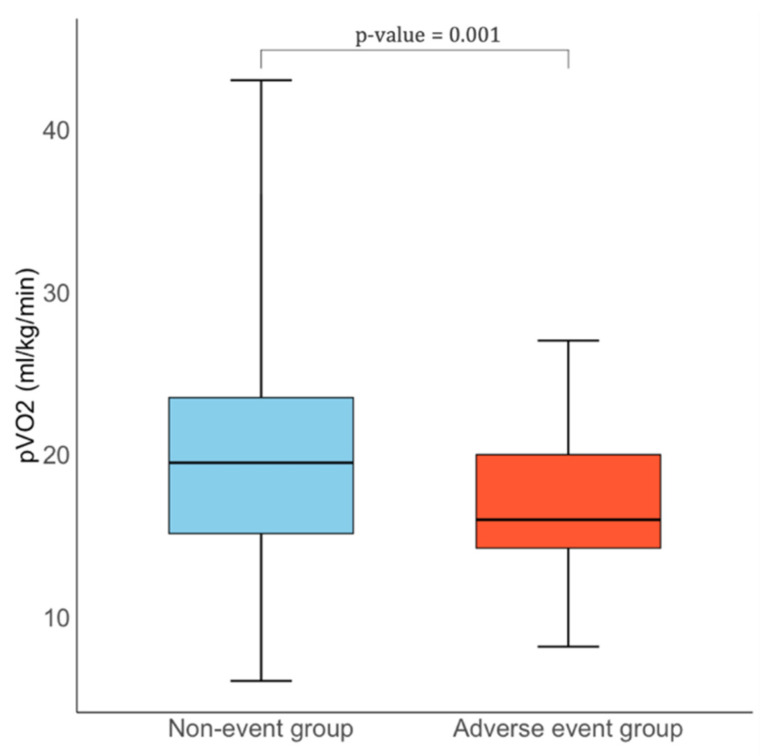
Comparison of pVO_2_ levels between HCM patients with and without adverse events.

CPET findings are summarized in Table 3. Patients with events had lower peak VO_2_ (16.0 [14.2–20.0] vs. 19.5 [15.1–23.5] mL/kg/min, *p* = 0.001) [Figure 1] and a reduced percentage of predicted peak VO_2_ (62.1% vs. 67.1%, *p* = 0.006). Cardiovascular efficiency was impaired, as reflected by a lower VO_2_ per Watt (8.2 [6.6–9.0] vs. 9.0 [8.0–10.4] mL/min/W, *p* = 0.026), while ventilatory efficiency was reduced with a higher VE/VCO_2_ slope (28.0 [25.0–33.0] vs. 27.0 [24.0–30.0], *p* = 0.048). Markers of submaximal performance were also altered: anaerobic threshold occurred earlier (7.0 [5.0–8.0] vs. 8.0 [6.0–10.0] min, *p* = 0.032), with lower oxygen uptake at AT (13.0 [11.1–15.0] vs. 16.0 [13.0–20.0] mL/kg/min, *p* < 0.001) and a reduced percentage of predicted AT (45.6% vs. 55.6%, *p* = 0.003).

### 3.4. Variables Associated with Clinical Events

Over a median follow-up of 7.4 [4.3–10.2] years, 63 patients (16.2%) experienced at least one clinically relevant adverse event, defined as heart failure hospitalization or progression to end-stage HCM. At univariate logistic regression (Table 4), age (OR 1.02, 95% CI 1.00–1.04, *p* = 0.048), dyslipidemia (OR 2.91, 95% CI 1.68–5.08, *p* < 0.0001), higher resting E/e′ (OR 1.09, 95% CI 1.04–1.14, *p* = 0.011), and lower peak VO_2_ (OR 0.91, 95% CI 0.87–0.96, *p* = 0.0006) were significantly associated with events. In multivariable analysis, dyslipidemia (OR 2.58, 95% CI 1.68–5.08, *p* = 0.006), higher resting E/e′ (OR 1.06, 95% CI 1.00–1.12, *p* = 0.042), and lower peak VO_2_ (OR 0.92, 95% CI 0.87–0.98, *p* = 0.016) remained independently associated with the primary outcome.

## 4. Discussion

This study demonstrates that combined assessment with cardiopulmonary exercise testing and stress echocardiography (CPET–SE) provides a comprehensive and synergistic evaluation of patients with HCM, capturing both the quantitative impairment in aerobic capacity and the pathophysiological mechanisms underlying exercise limitation.

From the echocardiographic standpoint, patients who experienced adverse events exhibited a more adverse structural and functional profile, with greater left atrial remodeling, more severe left ventricular hypertrophy, and evidence of impaired diastolic reserve. The rise in E/e′ during exercise, together with higher pulmonary artery systolic pressures, points to a reduced ability to accommodate exercise-related increases in preload and to impaired ventriculo–atrial coupling. These mechanisms may contribute to symptom development and long-term clinical deterioration. Importantly, these differences were observed despite similar LVEF and LVOT gradients at rest and during provocation, indicating that, in many cases, functional limitation was driven more by diastolic and atrial factors than by outflow obstruction [22].

CPET data aligned closely with these imaging findings. The adverse-event group showed markedly reduced peak VO_2_, earlier anaerobic threshold, impaired ventilatory efficiency, and signs of chronotropic incompetence. The parallel between diastolic dysfunction on stress echocardiography and reduced aerobic capacity suggests that elevated filling pressures during exertion may be a major determinant of exercise intolerance in this population. CPET offers a comprehensive and dynamic assessment of cardiovascular reserve. In contrast to conventional imaging modalities, which are largely static and load-dependent, CPET-derived parameters such as peak VO_2_ can unmask early physiological deterioration not evident at rest [23]. This is especially relevant in HCM, where patients often maintain preserved ejection fraction and minimal symptoms until advanced stages. Impaired peak VO_2_ reflects a cumulative deficit in diastolic filling, myocardial energetics, and microvascular perfusion that drive exercise intolerance [24].

Several large cohort studies have consistently shown that patients with peak VO_2_ values < 14 mL/kg/min or <50% of age- and sex-predicted values face substantially increased risks of progression to advanced heart failure or the need for heart transplantation [25]. For instance, Coats et al. demonstrated in nearly 1900 patients that a peak VO_2_ ≤ 15.3 mL/kg/min was associated with a 10-year event rate (death or transplant) exceeding 30% [26]. Similarly, Masri et al. reported that a lower percentage of predicted peak VO_2_ was independently associated with higher incidence of all-cause mortality, ICD shocks, and heart failure hospitalizations [8]. Our results corroborate and extend this evidence by showing that reduced peak VO_2_ remains strongly associated with clinically meaningful outcomes even when integrated within a multiparametric CPET–SE framework.

We also explored additional CPET parameters, including VE/VCO_2_ slope, oxygen pulse, and anaerobic threshold. While these variables were associated with adverse outcomes in univariate analyses, they did not remain independently associated in multivariable analysis. This is consistent with prior research indicating that, although a steep VE/VCO_2_ slope and early anaerobic threshold reflect ventilatory inefficiency and impaired haemodynamics, their prognostic capacity in HCM is generally inferior to that of peak VO_2_. The primacy of peak VO_2_ likely reflects its integrative nature, incorporating contributions from diastolic filling, myocardial energetics, microvascular perfusion, chronotropic response, and peripheral oxygen utilization [27].

On the echocardiographic side, our multivariable analysis confirmed that higher E/e′ at rest is independently associated with adverse events. The independent prognostic contribution of stress-derived diastolic indices reinforces the role of dynamic, rather than solely resting, measurements in risk stratification [28].

Another clinically relevant finding is the independent association between dyslipidemia and the composite endpoint. Although at first glance unexpected in a sarcomeric disease, this observation is biologically plausible and supported by emerging evidence. Observational studies have suggested that dyslipidemia may identify patients with blunted improvement in VO_2_ peak following septal reduction therapy, potentially reflecting adverse interactions between metabolic milieu, microvascular function, and myocardial energetics [29]. More broadly, metabolic dysregulation has been increasingly recognized as a modifier of HCM phenotype and trajectory [30]. Dyslipidemia should therefore be considered a modifiable risk factor and a pragmatic target for optimization while the field awaits high-quality interventional data.

From a clinical perspective, the combined CPET–SE approach enables simultaneous identification of functional limitation, hemodynamic abnormalities, and actionable therapeutic targets. In patients with reduced peak VO_2_ and stress-induced elevations in E/e′ and pulmonary pressures, closer surveillance and earlier, mechanism-oriented interventions may be warranted—ranging from aggressive risk-factor control (including lipid management), to pharmacologic strategies aimed at diastolic function and heart-rate control, to timely consideration of septal reduction in appropriately selected obstructive phenotypes [31]. Beyond individual parameters, the strength of CPET–SE lies in its integrated read-out: quantifying “how much” capacity is lost (via CPET) while clarifying “why” (via SE), thus refining risk stratification compared with either modality alone.

## 5. Strengths and Limitations

Key strengths of this study include the large, well-characterized cohort, the long follow-up, and the simultaneous application of multimodal diagnostics. Limitations include the observational, single-center design—potentially limiting generalizability—and the predominantly Caucasian population. A Cox proportional hazards model could not be applied because the exact timing of events was not consistently available, precluding accurate time-to-event estimation and censoring. Consequently, logistic regression was used to identify independent predictors of event occurrence: this approach captures overall associations but does not provide temporal risk estimates. Residual confounding cannot be excluded, and external validation in more diverse cohorts is warranted. Moreover, as an observational study, it cannot determine whether CPET–SE–guided management directly translates into therapeutic changes or improved outcomes, an issue that future prospective studies should address.

## 6. Conclusions and Clinical Implications

Our study demonstrates that CPET–SE offers a powerful and integrated approach to prognostic assessment in HCM. By linking objective measures of functional capacity with the hemodynamic mechanisms underlying exercise limitation, CPET–SE refines risk stratification beyond either modality alone. Reduced peak VO_2_ emerged as the strongest predictor of adverse outcomes, while abnormal stress-derived diastolic indices such as elevated E/e′ identified a distinct high-risk phenotype. These findings suggest that impaired exercise capacity should prompt careful evaluation of diastolic reserve, and that stress abnormalities may justify earlier, mechanism-oriented interventions even in patients with mild or borderline symptoms. Early recognition of this combined functional–hemodynamic impairment may support closer surveillance, timely pharmacologic or interventional treatment, and optimal referral for advanced heart failure therapies. Prospective studies are warranted to evaluate whether CPET–SE–guided management can improve clinical outcomes and help personalize long-term care in HCM.

## Figures and Tables

**Table 1:** Clinical characteristics.

	Overall (N = 388)	Non-Event Group (N = 325)	Adverse Event Group (N = 63)	*p*-Value
Age, years	48 ± 15	47 ± 15	51 ± 13.26	0.047
Male, n (%)	245 (63.1)	206 (63.4)	39 (61.9)	0.936
BSA, m^2^	1.86 ± 0.21	1.86 ± 0.21	1.87 ± 0.20	0.694
Comorbidities, n (%)				
Hypertension	153 (39.4)	119 (36.6)	34 (54.0)	0.015
Dyslipidaemia	117 (30.2)	85 (26.2)	32 (50.8)	<0.001
Diabetes Mellitus	30 (7.8)	24 (7.4)	6 (9.5)	0.751
COPD	7 (1.8)	6 (1.8)	1 (1.6)	1.000
PAD	9 (2.3)	6 (1.8)	3 (4.8)	0.342
CAD	19 (4.9)	14 (4.3)	5 (7.9)	0.367
Prior Stroke	12 (3.1)	9 (2.8)	3 (4.8)	0.661
AF	51 (13.1)	31 (9.5)	20 (31.7)	<0.001
Presenting symptoms, n (%)				
Chest pain	121 (31.3)	103 (31.8)	18 (28.6)	0.722
Dyspnoea	192 (49.5)	150 (46.2)	42 (66.7)	0.004
Palpitations	99 (25.5)	79 (24.3)	20 (31.7)	0.279
Syncope	52 (13.4)	45 (13.8)	7 (11.1)	0.703
NYHA functional class, n (%)				<0.001
I	179 (46.3)	166 (51.2)	13 (20.6)	
II	181 (46.6)	140 (43.1)	41 (65.1)	
III	25 (6.4)	17 (5.2)	8 (12.7)	
IV	3 (0.8)	3 (0.9)	0 (0.0)	
ESC SCD risk score for HCM	3.7 ± 2.4	3.5 ± 2.2	4.9 ± 2.6	<0.001
Medications, n (%)				
Aspirin	61 (15.7)	42 (12.9)	19 (30.2)	0.001
Beta-blockers	236 (60.8)	190 (58.5)	46 (73.0)	0.043
ACE inhibitors	68 (17.5)	44 (13.5)	24 (38.1)	<0.001
Angiotensin II receptor blockers	86 (22.2)	72 (22.2)	14 (22.2)	1.000
Anticoagulants	49 (12.7)	30 (9.3)	19 (30.2)	<0.001
Calcium channel blockers	32 (8.2)	26 (8.0)	6 (9.5)	0.879
ARNI	0 (0)	0 (0)	0 (0)	
Disopyramide	39 (10.1)	29 (9.0)	10 (15.9)	0.149
Amiodarone	33 (8.5)	20 (6.2)	13 (20.6)	<0.001
Ranolazine	14 (3.6)	10 (3.1)	4 (6.3)	0.365
MRA	14 (3.6)	6 (1.8)	8 (12.7)	<0.001
SGLT2 inhibitors	4 (1.0)	4 (1.2)	0 (0.0)	0.839
Diuretics	75 (19.4)	48 (14.9)	27 (43.5)	<0.001

AF = atrial fibrillation; ACE = angiotensin-converting enzyme; ARNI = angiotensin receptor-neprilysin inhibitors; BSA = body surface area; CAD = coronary artery disease; COPD = chronic obstructive pulmonary disease; ESC SCD Risk Score for HCM = European Society of Cardiology Sudden Cardiac Death Risk Score for Hypertrophic Cardiomyopathy; MRA = mineralocorticoid receptor antagonists; NYHA = New York Heart Association; PAD = peripheral arterial disease.

**Table 2:** Echocardiographic characteristics between groups.

	Overall (N = 388)	Non-Event Group (N = 325)	Adverse Event Group (N = 63)	*p*-Value
LA diameter, mm	41.5 [38.0, 46.0]	41.0 [37.0, 45.0]	45.0 [41.0, 52.0]	<0.001
LV maximal wall thickness, mm	20.0 [17.0, 23.0]	20.0 [17.0, 23.0]	22.0 [19.0, 25.0]	0.001
LVEF, %	66.0 [61.7, 72.5]	66.0 [62.5, 72.5]	66.3 [58.9, 71.7]	0.151
Resting/stress SAM, n (%)				0.022/0.346
Mild	102 (26.4)/71 (18.5)	84 (26.0)/ 56 (17.4)	18 (28.6)/ 15 (23.8)	
Moderate	47 (12.2)/39 (10.2)	45 (13.9)/ 36 (11.2)	2 (3.2)/ 3 (4.8)	
Severe	17 (4.4)/49 (12.8)	11 (3.4)/ 41 (12.8)	6 (9.5)/ 8 (12.7)	
LVEDV, mL	80.0 [65.0, 100.0]	80.0 [65.0, 100.0]	82.0 [64.0, 96.7]	0.850
LVESV, mL	26.0 [20.0, 35.0]	26.0 [19.0, 35.0]	25.50 [21.00, 36.00]	0.295
LVEDV index, mL/m^2^	44.8 [34.4, 52.0]	44.1 [35.3, 51.9]	45.3 [34.1, 53.5]	0.852
LVESV index, mL/m^2^	14.1 [10.5, 18.5]	14.0 [10.2, 18.4]	14.3 [11.3, 19.8]	0.344
Resting LA 4CH volume index	30.4 [24.0, 39.4]	29.0 [23.0, 37.0]	36.4 [29.8, 49.0]	<0.001
Stress LA 4CH volume index	27.5 [0.0, 49.7]	26.0 [0.0, 44.0]	43.0 [0.0, 66.0]	0.046
Resting/stress MR grade, n (%)				0.09/0.178
Grade I	161 (43.0)/115 (31.4)	126 (40.3)/ 92 (30.0)	35 (57.4)/ 23 (39.0)	
Grade II	24 (6.4)/33 (9.0)	20 (6.4)/ 27 (8.8)	4 (6.6)/ 6 (10.2)	
Grade III	8 (2.1)/16 (4.4)	7 (2.2)/ 11 (3.6)	1 (1.6)/ 5 (8.5)	
Grade IV	0 (0.0)/1 (0.3)	0 (0.0)/ 1 (0.3)	0 (0.0)/ 0 (0.0)	
Resting PASP, mmHg	20.0 [12.0, 28.0]	20.0 [11.5, 26.0]	25.0 [15.0, 32.0]	0.027
Stress PASP, mmHg	34.0 [25.0, 45.0]	34.0 [25.0, 45.0]	37.0 [25.7, 54.0]	0.044
Resting E/A	1.1 [0.8, 1.5]	1.08 [0.8, 1.5]	1.1 [0.8, 1.6]	0.613
Stress E/A	1.1 [0.8, 1.6]	1.1 [0.8, 1.5]	1.2 [0.9, 1.8]	0.105
Resting average E/e′	9.9 [7.9, 13.1]	9.8 [7.7, 12.6]	11.4 [9.0, 17.3]	0.004
Stress average E/e′	10.3 [8.3, 12.9]	9.9 [8.1, 12.3]	11.2 [9.6, 15.5]	0.001
Resting septal E/e′	11.8 [9.5, 16.0]	11.6 [9.3, 15.5]	13.4 [11.0, 20.0]	0.001
Stress septal E/e′	11.6 [9.5, 15.5]	11.5 [9.3, 15.0]	13.7 [11.3, 19.0]	<0.001
Resting lateral E/e′	8.0 [6.0, 11.0]	7.9 [5.9, 10.8]	9.4 [6.6, 13.7]	0.025
Stress lateral E/e′	8.6 [6.7, 11.0]	8.2 [6.7, 10.7]	10.0 [7.3, 13.6]	0.010
E wave, cm/s	73.0 [60.5, 90.0]	73.0 [62.0, 90.0]	69.0 [58.0, 83.7]	0.355
A wave, cm/s	66.0 [52.0, 84.0]	67.0 [53.0, 84.7]	60.0 [45.0, 77.2]	0.077
E wave peak velocity, cm/s	100.0 [85.0, 120.0]	100.0 [85.2, 120.0]	110.0 [82.7, 123.0]	0.485
A wave peak velocity, cm/s	89.0 [67.5, 115.0]	91.0 [68.2, 116.0]	80.0 [67.0, 109.0]	0.110
Resting TAPSE, mm	21.0 [19.0, 23.0]	21.0 [20.0, 23.0]	21.0 [19.00, 22.7]	0.317
Stress TAPSE, mm	25.0 [22.0, 28.0]	25.0 [22.0, 28.0]	24.5 [22.0, 27.0]	0.271
Resting LVOT gradient, mmHg	12.0 [8.0, 30.0]	11.0 [8.0, 30.0]	14.0 [8.0, 28.5]	0.527
Provoked LVOT gradient, mmHg	30.0 [19.0, 70.0]	30.0 [19.0, 70.0]	35.0 [19.5, 70.0]	0.749

LA = left atrium; LVEDV = left ventricular end-diastolic volume; LVEF = left ventricular ejection fraction; LVESV = left ventricular end-systolic volume; LVOT = left ventricular outflow tract; PASP = pulmonary artery systolic pressure; SAM = systolic anterior motion of the mitral valve; TAPSE = tricuspid annular plane systolic excursion; 4CH = 4-chamber view.

**Table 3:** CPET findings.

	Overall (N = 388)	Non-Event Group (N = 325)	Adverse Event Group (N = 63)	*p*-Value
Workload, watts	90.0 [70.0, 120.0]	97.5 [70.0, 120.0]	90.0 [60.0, 110.0]	0.015
Peak VO_2_, mL/kg/min	19.0 [15.0, 23.0]	19.5 [15.1–23.5]	16.0 [14.2–20.0]	0.001
% VO_2_ predicted	66.2 [55.6, 78.1]	67.1 [56.1, 80.5]	62.1 [48.1, 73.0]	0.006
VO_2_/Watt, mL/min/W	8.9 [7.7, 10.0]	9.0 [8.00 10.4]	8.2 [6.6, 9.0]	0.026
O_2_ pulse, VO_2_/HR	11.0 [8.6, 14.3]	11.0 [8.6, 14.2]	11.0 [8.9, 14.4]	0.960
VE/VCO_2_ slope	27.0 [24.0, 30.2]	26.6 [24.0, 30.0]	28.0 [25.0, 33.0]	0.048
Breathing Reserve, %	42.0 [32.0, 52.0]	42.4 [32.0, 53.0]	41.5 [33.5, 50.2]	0.793
AT, min	8.0 [6.0, 9.0]	8.0 [6.0, 10.0]	7.0 [5.0, 8.0]	0.032
% AT predicted	54.8 [42.7, 64.8]	55.6 [44.3, 66.1]	45.6 [34.5, 61.9]	0.003
AT, mL/kg/min	15.1 [12.6, 19.0]	16.0 [13.0, 20.0]	13.0 [11.1, 15.0]	<0.001
HR rest, bpm	74.0 [65.0, 86.0]	75.0 [65.0, 87.0]	69.0 [63.0, 78.0]	0.003
HR max, bpm	128.0 [110.0, 144.0]	130.0 [112.0, 146.0]	114.0 [101.5, 129.0]	<0.001
% HR predicted	79.0 [70.0, 88.0]	81.0 [71.0, 90.0]	73.0 [64.0, 80.5]	<0.001
Systolic BP rest, mmHg	120.0 [110.0, 140.0]	120.0 [110.0, 140.0]	120.0 [110.0, 140.0]	0.831
Systolic BP peak, mmHg	170.0 [150.0, 190.0]	170.0 [150.0, 190.0]	160.0 [142.5, 187.5]	0.051
Diastolic BP rest, mmHg	80.0 [70.0, 85.0]	80.0 [70.0, 85.0]	75.0 [70.0, 80.0]	0.050
PETCO_2_ rest, mmHg	37.0 [32.0, 41.2]	37.0 [32.0, 41.0]	38.0 [35.0, 43.7]	0.254
PETCO_2_ peak, mmHg	42.0 [36.0, 46.0]	41.0 [36.0, 46.0]	42.0 [39.2, 46.0]	0.499

AT = anaerobic threshold; BP = blood pressure; CPET = cardiopulmonary exercise testing; HR = heart rate; PETCO_2_ = partial pressure of end-tidal carbon dioxide; VE/VCO_2_ = ventilatory equivalent for carbon dioxide; VO_2_ = oxygen consumption.

**Table 4:** Univariate and multivariate logistic regression analysis for predictors of adverse events in patients with HCM.

Variable	Univariate Regression		Multivariate Regression	
	OR (95% CI)	*p*-Value	OR (95% CI)	*p*-Value
Age (per year)	1.02 (1.00–1.04)	0.048	0.98 (0.96–1.01)	0.162
Gender	0.94 (0.54–1.65)	0.824	-	-
Dyslipidaemia	2.91 (1.68–5.08)	<0.0001	2.58 (1.68–5.08)	0.006
Diabetes Mellitus	1.32 (0.47–3.17)	0.567	-	-
Resting average E/e′	1.09 (1.04–1.14)	0.011	1.06 (1.00–1.12)	0.042
pVO_2_	0.91 (0.87–0.96)	0.0006	0.92 (0.87–0.98)	0.016
O_2_ pulse, VO_2_/HR	1.00 (0.94–1.07)	0.957	-	-
VE/VCO_2_ slope	1.03 (0.99–1.08)	0.145	-	-
PETCO_2_ peak, mmHg	1.03 (0.96–1.11)	0.493	-	-

## Data Availability

The data presented in this study are available on request from the corresponding author. The data are not publicly available due to privacy restrictions.

## Abbreviations

The following abbreviations are used in this manuscript:

HCM	Hypertrophic Cardiomyopathy
CPET	Cardiopulmonary Exercise Testing
SE	Stress Echocardiography
VO_2_	Oxygen Consumption
VE/VCO_2_	Ventilatory Equivalent for Carbon Dioxide
AT	Anaerobic Threshold
HR	Heart Rate
BP	Blood Pressure
PETCO_2_	Partial Pressure of End-Tidal Carbon Dioxide
LVOT	Left Ventricular Outflow Tract
LVEF	Left Ventricular Ejection Fraction
LA	Left Atrium
LV	Left Ventricle
LVEDV	Left Ventricular End-Diastolic Volume
LVESV	Left Ventricular End-Systolic Volume
NYHA	New York Heart Association
ESC	European Society of Cardiology
SCD	Sudden Cardiac Death
ICD	Implantable Cardioverter Defibrillator
CAD	Coronary Artery Disease
CMR	Cardiac Magnetic Resonance
AF	Atrial Fibrillation
COPD	Chronic Obstructive Pulmonary Disease
PAD	Peripheral Artery Disease
BSA	Body Surface Area
ACE	Angiotensin-Converting Enzyme
ARNI	Angiotensin Receptor–Neprilysin Inhibitor
MRA	Mineralocorticoid Receptor Antagonist
SGLT2	Sodium-Glucose Cotransporter 2
SAM	Systolic Anterior Motion (of the mitral valve)
PASP	Pulmonary Artery Systolic Pressure
TAPSE	Tricuspid Annular Plane Systolic Excursion
4CH	Four-Chamber View
MR	Mitral Regurgitation
